# New therapeutic hypothesis for infantile extrinsic hydrocephalus

**DOI:** 10.3389/fneur.2023.1215560

**Published:** 2023-09-19

**Authors:** Masahiro Kameda, Yoshinaga Kajimoto, Masahiko Wanibuchi

**Affiliations:** Department of Neurosurgery, Osaka Medical and Pharmaceutical University, Takatsuki, Japan

**Keywords:** pediatric hydrocephalus, glucagon-like peptide-1 (GLP-1), erythropoietin (EPO), free radical scavenger, post-hemorrhagic hydrocephalus

## Current standard therapy for hydrocephalus

Cerebrospinal fluid (CSF) shunting, as exemplified by the ventriculoperitoneal (VP) shunt, is the gold standard for the treatment of hydrocephalus in both adults and children. In fact, the SINPHONI and SINPHONI-2 studies demonstrated that shunting for idiopathic normal pressure hydrocephalus (iNPH) is medically and economically beneficial due to its therapeutic effect (1, 2). However, the short- and long-term complications of shunt surgery for hydrocephalus remain unresolved. For example, shunt failure is reported to occur in up to 40% of cases within the 1st year after surgery (3–6). Shunt infections have not been completely eliminated, although they are less common than in the past (7–9).

Furthermore, especially pediatric cases, it is indisputable that the long-term psychosocial burden associated with the shunt system affects the quality of life, as the shunt system is required for the rest of the patient's life.

## Current status of hydrocephalus treatment research

The VP shunt, the standard treatment for hydrocephalus, is a countermeasure to bring a progressive and worsening state of hydrocephalus into a state of arrested hydrocephalus. Shunt surgery is already an established treatment for hydrocephalus, and many outcomes have been reported for the shunt itself (10–12).

In contrast, because shunt surgery requires permanent implantation of the shunt system, surgical techniques that do not require permanent implantation, such as endoscopic third ventriculography (ETV) and choroid plexus coagulation (CPC), have been developed. Some reports have compared the therapeutic efficacy of these techniques with that of shunting (13–16). Simply stated, ETV is more effective for obstructive hydrocephalus than non-obstructive hydrocephalus, and has a therapeutic effect for obstructive hydrocephalus comparable to shunting. In addition, the combination of ETV and CPC has been tried in non-obstructive hydrocephalus, but the results have not been satisfactory, while shunting is effective in both non-obstructive and obstructive hydrocephalus. Therefore, the combination of ETV and CPC is not a common treatment option at this time.

Failure to control progressive neurologic deterioration and ventricular enlargement on imaging requires shunt placement. However, there is insufficient research to develop histopathology-based treatments that prevent progressive deterioration and avoid shunt surgery.

In routine practice, a number of patients have enlarged ventricles on imaging but no obvious symptoms of hydrocephalus. Furthermore, before closure of the anterior fontanel in infants, intracranial hypertension may, for some time, be compensated for by an increase in head circumference, even with ventricular enlargement (17).

Although basic and translational histopathology-based research for central nervous system (CNS) diseases such as stroke and Parkinson's disease has been well-established for future clinical application (18–21), histopathology-based treatments for hydrocephalus have not been adequately explored.

If restoring the microenvironment in the brain could prevent the progression of hydrocephalus to a condition requiring surgery, it would mark the beginning of a new era in the treatment of infantile extrinsic hydrocephalus, such as post-hemorrhagic hydrocephalus from shunt surgery to shunt avoidance.

## Importance of proper discharge and removal of hazardous materials

To maintain neurological activity, waste products and other toxic substances must be properly eliminated from the brain. Lymphatic vessels play this role in other organs, but a similar system in the CNS had not been identified. In 2012, the glymphatic system was proposed as a pathway to transport and drain substances into the subarachnoid space of the brain via astrocytes. This pathway was found to be dependent on aquaporin-4 (AQP4), a water channel protein in astrocytes (22).

It has now been established that the glymphatic system in the brain plays a role analogous to that of the lymphatic system in the body, efficiently removing waste products to the outside of the brain by generating extracellular flow.

In recent years, we have gained new information about the physiology of the cerebrospinal fluid, including the glymphatic system, the paravascular space, and the interstitial fluid (22–26), and we believe that this is a good time to develop new treatment and management methods for hydrocephalus based on this new knowledge.

## Development of a new treatment incorporating the glymphatic system theory: Alzheimer's disease

Recently, it was reported in a mouse model that when the removal of extracellular tau protein by the glymphatic system is inhibited, the amount of tau in the brain increases, affecting neurodegeneration. AQP4 is involved in this clearance process, and mice lacking AQP4 showed increased tau accumulation and neuronal cell death (27).

In addition, the greater the dysfunction of the glymphatic system, the less amyloid-β is found in the CSF; similarly, the more that amyloid-β is unable to be cleared from the CSF, the greater the deposition of amyloid-β in the brain (28).

Thus, delayed clearance of Alzheimer-related proteins due to dysfunction of the glymphatic system has been reported to contribute, at least in part, to the development of Alzheimer's disease.

In addition to hydrocephalus, at least some cases of chronic fatigue syndrome have been reported to benefit from CSF drainage, leading to speculation that accumulation of toxic substances in the CNS due to glymphatic dysfunction may be involved (29).

## Improvement of hydrocephalus is expected by improving inflammatory findings in the ventricular and paraventricular microenvironment

Intraventricular hemorrhage in preterm babies is caused by perforation of the ventricle in neonates with germinal matrix layer hemorrhage, and neuroinflammation in the paraventricular tissue is reportedly involved in the pathogenesis of this condition. This inflammatory milieu generates free radicals and pro-inflammatory cytokines such as interleukin (IL)-6, IL-4, tumor necrosis factor-α (TNFα), and transforming growth factor-β1 (TGFβ1), which contribute to the development and progression of hydrocephalus (30).

According to the osmotic gradient theory, brain diseases with excess macromolecules in the intracerebroventricular spinal fluid alter the osmotic gradient and cause hydrocephalus. In other words, hydrocephalus can be considered a macromolecular clearance disorder rather than a circulatory disorder (23).

AQPs, known water transport proteins, are transmembrane water channel, and the direction of water transport by AQP follows only an osmotic gradient; that is, AQPs are passive water transport proteins (25).

Although the relationship between the CSF in the ventricles and the glymphatic system is not well-understood, effective removal of the toxic macromolecular proteins underlying the pathogenesis of hydrocephalus may prevent glymphatic dysfunction, prevent neurological deterioration, ventricular enlargement, and ultimately shunting. Furthermore, based on the osmotic gradient theory, effective removal of the toxic macromolecular proteins underlying the pathogenesis of hydrocephalus may prevent progressive neurological deterioration and ventricular enlargement on imaging thus avoiding shunt surgery. Regardless of whether it follows the osmotic gradient theory or the glymphatic system theory, AQP is considered to play a key role.

## Development of new treatment and management methods for hydrocephalus

To develop a new treatment and management of hydrocephalus that prevents the transition to hydrocephalus requiring a shunt, it is necessary to improve the inflammatory environment through efficient removal of toxic macromolecular proteins and to improve the ventricular and paraventricular microenvironment.

The question is how to effectively remove toxic macromolecular proteins. There is a history of developing new treatments for CNS diseases in terms of scavenging free radicals and improving the microenvironment. For example, edaravone was initially approved for cerebral infarction but the indication was later expanded to include amyotrophic lateral sclerosis (ALS) (31).

In the human brain, neurogenesis has been observed in the subventricular zone and the hippocampus. Given the background pathology of hydrocephalus, improving the ventricular and paraventricular microenvironment is expected to be effective in preserving the function of neurogenesis, which is thought to be innate in the human brain (32). If microenvironmental repair can prevent hydrocephalus from progressing to the state where it requires surgery, it will usher in a new era in infantile extrinsic hydrocephalus management, from surgery to prevention.

The glucagon-like peptide-1 (GLP-1) receptor drug liraglutide (33, 34) and erythropoietin (EPO) (35, 36) have been shown to promote neurogenesis and to have anti-inflammatory properties. GLP-1 receptor agonists are drugs used to treat patients with diabetes without the risk of hypoglycemic events, and their ability to enhance neurogenesis and anti-inflammatory effects is attractive for application in the treatment of hydrocephalus (37). In addition, although EPO has a side effect of polycythemia, carbamoylated erythropoietin (CEPO), a neuroprotective agent without the risk of polycythemia, has been developed, and its ability to enhance neurogenesis and anti-inflammatory effects is attractive when considering its application in the treatment of hydrocephalus (38–41).

In terms of the osmotic gradient and glymphatic system theories, AQP4 has been shown to play an important role in the efficient removal of toxic macromolecular proteins; moreover, EPO upregulates AQP4 expression and improves the clearance of excess water via AQP4 (42). Moreover, exenatide, a GLP-1 passive agonist similar to liraglutide, has been reported to restore reduced AQP4 levels in the hippocampus of diabetic rats (43).

In this context, cocktail therapy with edaravone, liraglutide and EPO is expected to prevent progressive neurological deterioration and ventricular enlargement, thus avoiding shunt surgery. In addition, if enhanced neurogenesis leads to recovery of neurological function, it is expected to lead to a new treatment that improves the functional prognosis of even surgically treated cases of hydrocephalus.

## Author contributions

MK contributed to conception and design of the study and wrote the manuscript under supervision of YK and MW. All authors contributed to manuscript revision, read, and approved the submitted version.
